# In Situ Ellipsometry Measurements on the Halide Phase Segregation of Mixed Halide Lead Perovskites**

**DOI:** 10.1002/cphc.202200022

**Published:** 2022-06-07

**Authors:** Annik Bernhardt, Tharushi D. Ambagaspitiya, Martin E. Kordesch, Katherine Leslee A. Cimatu, Jixin Chen

**Affiliations:** ^1^ Department of Chemistry and Biochemistry Ohio University Athens OH 45701 United States; ^2^ Department of Chemistry University of Leipzig 04103 Leipzig Germany; ^3^ Nanoscale and Quantum Phenomena Institute Ohio University Athens OH 45701 United States; ^4^ Department of Physics and Astronomy Ohio University Athens OH 45701 United States

**Keywords:** mixed-halide lead perovskites, phase separation, ellipsometry, kinetic measurements, solar cells

## Abstract

Methylammonium lead iodide bromides MAPb(Br_x_I_1‐x_)_3_ are a class of mixed halide lead perovskites, materials that offer high‐power conversion efficiencies and bandgap tunability. For these reasons, they are a promising absorber material for future solar cells, although their use is still limited due to several factors. The reversible phase segregation under even low light intensities is one of them, lowering the effective bandgap due to local segregation into iodide‐rich and bromide‐rich phases. While several studies have been done to illuminate the mechanism and suppression of phase segregation, challenges remain to understand its kinetics. We obtained dynamic ellipsometric measurements from *x*=0.5 mixed halide lead perovskite thin films protected by a polystyrene layer under green laser light with a power density of ∼11 W/cm^2^. Time constants between 1.7(±0.7)×10^−3^ s^−1^ for the segregation and 1.5(±0.6)×10^−4^ s^−1^ for recovery were calculated. The phase segregation rate constants are surprisingly two orders of magnitude slower than and the recovery rate is consistent with those measured using photoluminescence methods under similar conditions. These results confirm a concern in the literature about the complexity in the phase separation kinetics measured from photoluminescence. We expect ellipsometry to serve as a complementary technique to other spectroscopies in studying mixed‐halide lead perovskites phase segregation in the future.

## Graphical abstract

**The phase segregation** of a mixed halide perovskite, a promising solar cell material, in iodide‐rich and bromide‐rich phases under laser light is tracked via dynamic ellipsometric measurements. The method allows for continuous tracking of sample composition. Calculated kinetic rate constants are consistent with literature values from absorption measurements.

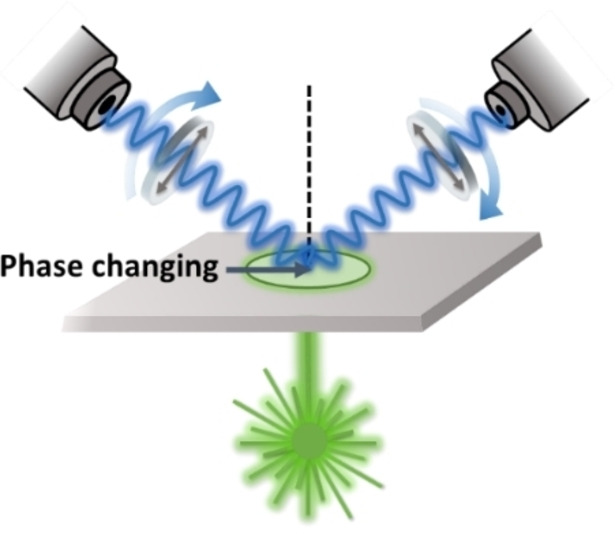

## Introduction

With power conversion efficiencies reaching up to 25.5 %, perovskites have emerged as a promising material for the next generation of solar cells.^[1]^ Mixed halide perovskites offer, among others, the advantages of good bandgap tunability.^[2]^ Hoke et.al. observed reversible phase segregation in mixed bromide and iodide methylammonium lead perovskites MAPb(Br*_x_* I_1‐*x*_)_3_ with a bromide content between 20 % and 100 % under illumination with light of various wavelengths and less than one sun intensity. They theorized the formation of small, iodide‐rich domains.^[2]^ Since then there have also been studies looking into the factors influencing phase segregation, its mechanism, and its influence on solar cell performance, which have been reviewed elsewhere.[^3^, ^4^, ^5^, ^6^, ^7^, ^8^, ^9^, ^10^, ^11^, ^12^]

There have been difficulties to quantify the phase segregation of mixed halide perovskites. From photoluminescence (PL) measurements which are most commonly employed, an estimation of the amounts of bromide‐rich and iodide‐rich phases are not easily available, since charge carriers rapidly transfer to the I‐rich phase that dominates the PL spectrum. The region with the highest iodide content will have the smallest bandgap and acts as a charge carrier trap. They have also been theorized to have a higher luminescence efficiency. The dominance of the I‐rich region in the PL spectrum obscures the composition of the other phases and the amount of each phase, as well as the kinetics of phase segregation due to the additional processes influencing the intensity of PL.[^2^, ^13^, ^14^]

Hoke et al. estimated the amount of iodide‐rich phase (*x*=0.2) to be 1 % from the photocurrent spectroscopy measurements of a mesoporous film in a solar cell device with 0.6 as the initial *x*. But x‐ray diffraction (XRD) measurements of a thin film with the same composition yield 23 % iodide‐ich phase, the rest being a single majority phase with *x*=0.7.^[2]^ XRD measurements were also carried out by Duong et al. who observed segregation of *x*=0.48 into *x*=1 and *x*=0.45 with 90 % of iodide‐rich phase.^[15]^ Barker et al.^[14]^ observed the phase segregation with photothermal deflection spectroscopy and arrived at values of 5 % for the iodide‐rich phase for both *x*=0.4 and *x*=0.6 thin‐film samples. Yoon et al.^[13]^ observed the segregation of *x*=0.43 by absorption measurements with 18 % of segregation into *x*=0.32 and *x*=1 estimated from a decrease in the absorption of the parent perovskite. A much lower amount of 2 % of segregation estimated from an increase in absorption for the I‐rich phase was explained by distorting effects of tail absorption. Several studies have concluded that besides the I‐rich and Br‐rich regions, a large part of the sample remains unchanged at the original composition.[^16^, ^17^]

Typically, the phase segregation results in an exponential growth of the photoluminescence intensity for the I‐rich phase and an exponential decay of the absorption of the original phase respectively, that saturates at a certain point. The rate constants of segregation range from 0.1–0.01 s^−1^ for photoluminescence[^2^, ^14^, ^18^] measurements and one magnitude smaller for absorption measurements.[^13^, ^19^, ^20^] The rate constants of the recovery range from 10^−2^ to 10^−5^ s^−1^.[^13^, ^14^, ^20^] These constants are also dependent on the excitation wavelength and intensity, as well as film thickness and quality.[^3^, ^4^, ^5^, ^6^, ^7^, ^8^, ^9^, ^10^, ^11^, ^12^]

Ellipsometry is an optical technique that measures the dielectric properties of thin films via the change in polarization upon reflection of light by a sample. This is usually expressed as two angles *Ψ* and *Δ* according to equation (1) with *R_p_* and *R_s_* being the reflection coefficients (the ratio of the respective vector components before and after reflection for the given direction) for two components perpendicular to the traveling direction of the light wave. The parameter *Ψ* describes the ratio of the amplitudes of p‐ and s‐polarized components, while *Δ* describes the phase shift between them.^[21]^

(1) $$\frac{R_{p}}{R_{s}}=tan\left(\Psi \right)e^{-i\Delta }$$

*Ψ* typically exhibits interference oscillations in thin‐film samples. In our study, a peak at the bandgap of the original perovskite is of interest, where changes due to the composition of the sample are most noticeable. The change to *Ψ* can be explained by shifts in the dielectric function of the material, which are particularly pronounced at the bandgap and correlate with the first peak of the dielectric function. (see also Supporting Information Figure S1 for the dielectric functions of the compositions used).^[21]^

Ellipsometry has previously been employed in the study of lead halide perovskites as reviewed recently by Li et.al.^[22]^ The method offers several advantages including fast, non‐destructive measurements that are sensitive to optical constants and the thickness of layered samples. Because it measures the reflective differences among polarized light at various wavelengths and angles, it is generally insensitive to disturbances by environmental light. The method is accurate, robust, reproducible, and can be sensitive to changes in phase, bandgap, and thickness.^[21]^ For this reason, phase segregation of perovskite samples protected by a polymer film can be observed under atmospheric conditions. Several phenomena of halide lead perovskites have been studied with ellipsometry previously. Optical functions and Tauc‐Lorentz‐Parameters for various MAPb(Br*_x_* I_1‐*x*_)_3_ perovskites have been tabulated by Fujimoto et.al.^[23]^ Ndione et.al.^[24]^ studied both complex optical and electrical functions of methylammonium, formamidinium (FA), and formamidinium−caesium mixed halide perovskites, additionally providing a critical point analysis. They found that substitution of halide had a bigger effect on optical functions than that of the A‐site cation. Similar optical function analysis has been carried out for other mixed perovskite systems such as by Ghimire et.al.^[25]^ for (FASnI_3_)_1‐x_(MAPbI_3_)_x_, Werner et.al.^[26]^ for Cs_y_FA_1‐y_Pb(I_x_Br_1‐x_)_3_, and Tejada et.al.^[27]^ for [FA_0.83_Cs_0.17_Pb(I_1‐x_Br_x_)_3_]. Degradation mechanisms and other phase changes have been studied as well. The light‐induced degradation of MAPbI_3_ solar cells was studied via ellipsometry and electrical measurements and additionally validated by XRD and DFT simulations by Maronnier et.al.^[28]^ They modeled the perovskite layer with several layers of Bruggemann Effective Medium approximations (EMA) containing differing percentages of voids and from the sixth day onward PbI_2_. The degradation mechanism of MAPbI_3_ single crystals and thin films, from hydration due to moist air on a timescale of several hours was studied by Leguy et.al..^[29]^ They used an EMA of the original perovskite and the corresponding monohydrate, as well as a surface roughness layer, and corroborated the results by XRD. Additionally, the mechanism was transferred to solar cells fabricated from the same perovskite. Similarly, the growth of co‐evaporated MAPbI_3_ including several segregated interface layers has been tracked with in‐situ ellipsometry by Ghimire et.al.^[30]^ Similar techniques have also been employed to determine optical functions of lead halide perovskites corrected by the surface roughness, presence of voids, or contaminants by several groups.[^31^, ^32^, ^33^, ^34^] To the best of our knowledge, no study on the light‐induced phase segregation of mixed halide perovskites using ellipsometric measurements has been done so far.

In the following communication, we present the observation of halide phase segregation with dynamic ellipsometric measurements. This method enables continuous measurement of the change in the composition during the segregation and recovery process.

## Results and Discussion

Perovskite thin films were synthesized via solvent‐washing, spin‐coating, and encapsulating with polystyrene with its protocol described elsewhere.^[32]^ The as‐synthesized mixed halide perovskites with *x*=0.1, *x*=0.5, and *x*=1 were characterized by both photoluminescence and UV‐Vis spectroscopy (Figure S2). The bandgap of samples with *x*=0.5 is ca. 665 nm (1.86 eV), the emission peak is at 656±5 nm and starts splitting into two peaks within 3 seconds of illumination. After 30 min of illumination, the photoluminescence peak of *x*=0.5 has shifted to 740±2 nm, a value close to samples with a nominal composition *x*=0.1 with a peak at 744±3 nm.

Samples with *x*=0.5 were further characterized. Scanning electron microscopy measurements (Figure S3) show uniform samples with some pinholes mainly below 10 nm but up to 80 nm in size. The grain size was estimated to be 56±13 nm (Figure S3). An XRD spectrum in Figure S4 shows expected 110 and 200 plane peaks. Ellipsometric measurements of the samples as exemplified in Figure S5 have revealed thicknesses of 195±4 nm for the perovskite layer and 1042±14 nm for the polystyrene layer. Average values of all fitting parameters for the three samples are given in Table S2. A control sample on Si substrate had a thickness of 869 nm and 193 nm for polystyrene and perovskite layers respectively, with a thickness non‐uniformity of 7 % as determined by the ellipsometric model fitting. Atomic force microscopy measurements (Figure S6) of the same sample showed consistent corresponding thicknesses of 825±48 nm and 199±19 nm respectively. Thickness non‐uniformity estimations ranged from 2 %–9 % for the polystyrene layer and 5 %–10 % for the perovskite layer.

**Figure 1:**
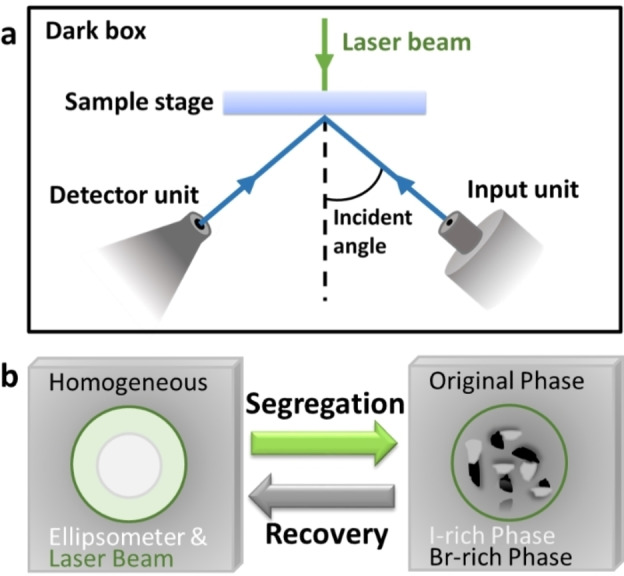
(a) Scheme of the ellipsometric measuring setup with safety box and (b) scheme of the phase segregation. The white spot represents the ellipsometry illumination light and the green spot represents the laser illumination light with a diameter of ∼2.5 mm.

**Figure 2:**
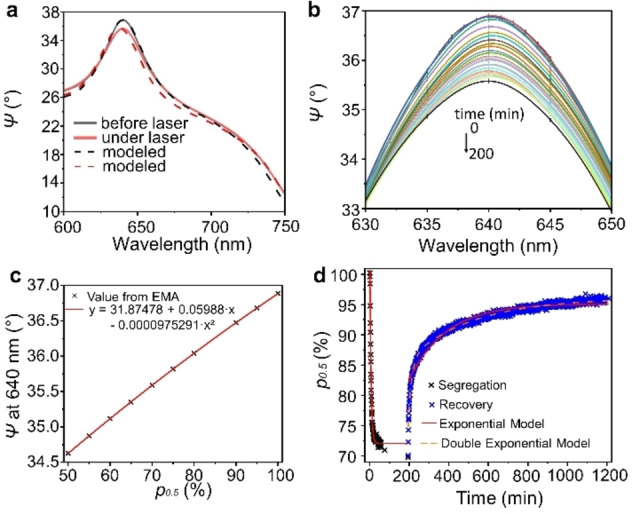
Ellipsometric amplitude parameter *Ψ* of *x*=0.5 at 50° (a) before laser exposure and under laser light including modeling by fitting algorithm, (b) relevant peak at selected times under laser exposure (peaks are interpolated between measurement points by Akima Splines, time labeled in Figure S7), (c) The dependence of the calculated value of *Ψ* on the content of *p*_0.5_ in a mixture of perovskite phases are fitted with a quadratic function, (d) segregation and recovery illustrated by the fraction of *x*=0.5 phase *p*_0.5_ over time and fitted by exponential functions.

Laser light shining through the backside of *x*=0.5 samples spun‐coat on glass coverslips initiated phase segregation. The power density of the laser illumination is estimated to be 11 W/cm^2^. The setup and separation scheme are provided in Figure 1. The laser beam was aimed such that it is focused on the same spot as the ellipsometer beam. The change in composition leads to the peak in the amplitude signal of ellipsometry (*Ψ*) within the bandgap region to decrease and shift (Figure 2a and 2b) most prominently at 50° and between 600 and 650 nm depending on sample thickness (Figure S7). In a control experiment, ellipsometric measurements of samples with composition *x*=1 and *x*=0.1 under the laser light were taken. Both had negligible changes (Figure S9) of *Ψ*. Changes in temperature, light scattering, or the polymer films should affect these samples similarly to samples with *x*=0.5. Thus, combining with results showing that these reference samples do not undergo phase changes or degradation, we conclude that the change in the ellipsometric measurements for *x*=0.5 is due to the phase segregation.[^2^, ^13^, ^14^, ^15^, ^17^, ^35^, ^36^]

The resulting mixture of phases during segregation and recovery of *x*=0.5 samples was modeled by a Bruggemann EMA of *x*=0.1, *x*=0.5, and *x*=1. The percentage amount of each phase *p_x_* was calculated assuming the decreased *x*=0.5 portion is transferred to *x*=1 and *x*=0.1 with equations (2) and (3) based on the stoichiometry of the phase segregation:

(2) $$p_{1}=\frac{\left(0.5-0.1\right)\cdot \left(100\;\%-p_{0.5}\right)}{1-0.1}$$

(3) $$p_{0.1}=100\;\%-p_{1}-p_{0.5}$$

While a variety of phases and a gradient should exist in the perovskite during the segregation process, an approximation using the original and two end‐point phases is feasible for this modeling approach and has been indicated by studies using other techniques.[^13^, ^15^] Here the composition of the iodide‐rich phase was determined from PL measurements. As mentioned previously, the composition of the bromide‐rich phase cannot be observed by PL. Previous studies using XRD^[15]^ and absorption spectroscopy^[13]^ measurements have indicated an end‐point bromide‐rich composition of *x*=1 for samples with similar initial compositions as used here, while others have indicated no bromide‐rich phase at all[^16^, ^17^] or a slight shift in the composition of the entire original phase.^[2]^ It should be noted that using only the original and iodide‐rich phase, besides being physically impossible, does not yield sensible fits since the observed peak in *Ψ* increases with increasing segregation instead of the experimentally observed decrease.

The predicted value of *Ψ* at the observed wavelength using the EMA model was fit to *p*_0.5_ between 100 % and 50 % with a quadratic function (Figure 2c). This calculation is carried out for each measurement using the initial spectrum time t=0 as the calibration point This also serves to remove any possible effect of roughness and interface variations among different samples with the same or different Br contents, i. e. the kinetics observed will be only from the change of the phases of the same sample rather than from other effects. From this data treatment, a decrease of *p*_0.5_ under laser light from 100 % to 77 % ±4% during measurement times ranging from 100–200 minutes was observed. This corresponds to 10 % of the bromide‐rich phase and 13 % of the iodide‐rich phase. Nine recovery measurements between 1000 and 1500 min in the dark revealed an increase of *p*_0.5_ to 99 % ±2%. The existence of endpoints above 100 % is from the error of the measurements themselves.

The segregation and recovery processes were fit by exponential decay and growth functions respectively. The time constants were found to be 1.7(±0.7)×10^−3^ s^−1^ for the segregation process and 1.5(±0.6)×10^−4^ s^−1^ for the recovery. One exemplary measurement with fitting is shown in Figure 2d. For the values of all measurements see Tables S3, S4, and S5 in the Supporting Information. The recovery kinetics is poorly fitted by the single exponential decay function. A double‐exponential curve fits all but one of the curves better indicating a more complicated recovery mechanism with an amplitude‐averaged rate constant calculated as 2.01(±1.26)×10^−3^ s^−1^. The parameters of these fits are listed in Table S6. Possibly there are multiple competing processes governing the recovery process and more research is needed to elucidate their mechanisms and relative strengths. The phase segregation rates are up to two orders of magnitude slower than those measured with photoluminescence[^2^, ^14^, ^18^] but more congruent with those determined by absorption measurements^[20]^ and the recovery rates are consistent with the literature values.[^19^, ^20^] These results confirm the concerns in previous studies[^2^, ^13^, ^14^] that because the PL is dominated by the I‐rich phase, the rate constants of phase separation obtained from these measurements are distorted by additional energy transfer processes. Combining different methods could provide future opportunities to decouple the phase separation and energy transfer processes.

## Conclusions

In summary, ellipsometry offers a simple and non‐destructive method to quantify the kinetics of phase segregation and the amount of the segregated phases without problems regarding quantum efficiency in PL measurements or shape and amorphous effects in XRD measurement. Thus, we expect it to serve as a complementary technique to other spectroscopies in studying mixed‐halide lead perovskites phase segregation. It cannot be completely excluded that the ellipsometry measurements are affected by changes to the morphology of the sample e. g. the formation of voids or changes to thickness non‐uniformity. Nevertheless, the relatively low noise and complete recovery (within 2 % error) of original measurements demonstrate its stability in quantifying phase changes.

## Experimental Section

Details of the experimental process are in the supporting information.

## Conflict of interest

The authors declare no conflict of interest.

1

## Supporting information

As a service to our authors and readers, this journal provides supporting information supplied by the authors. Such materials are peer reviewed and may be re‐organized for online delivery, but are not copy‐edited or typeset. Technical support issues arising from supporting information (other than missing files) should be addressed to the authors.

Supporting Information

## Data Availability

The data that support the findings of this study are available from the corresponding author upon reasonable request.
