# Supplementary material for: In Situ Ellipsometry Measurements on the Halide Phase Segregation of Mixed Halide Lead Perovskites
Source: Chemphyschem. 2022 Jun 7;23(15):e202200022. doi: 10.1002/cphc.202200022 (PMC9401859; doi:10.1002/cphc.202200022)
Supplement: Supplementary file 1 — Supporting Information [file CPHC-23-0-s001.pdf]

ChemPhysChem

Supporting Information

In Situ Ellipsometry Measurements on the Halide Phase Segregation of Mixed Halide Lead Perovskites**

Annik Bernhardt, Tharushi D. Ambagaspitiya, Martin E. Kordesch, Katherine Leslee A. Cimatu, and Jixin Chen*

Supporting Information

I

Table of Contents

Experimental Methods S2
Materials S2
Methods S2
Synthesis S2
Ellipsometry Measurements S2
Additional Measurements S3
Supplementary Figures and Tables S4
References S8

Experimental Methods

Materials

All chemicals were used without purification. Dimethyl sulfoxide (anhydrous, >99.9%), toluene (anhydrous, 99.8%), ethyl acetate (anhydrous, 99.8%), lead bromide (99.999%, PbI_2), and polystyrene (M_w: 280 000) were purchased from Sigma-Aldrich Chem. Co. (St. Louis, MO USA). Dimethylformamide (extra dry, 99.8%) was purchase from Acros Organics™. Methylammonium iodide (MAI) and Methylammonium bromide (MABr) were procured from GreatCellSolar (Queanbeyan, NSW Australia), Lead iodide (99.9985%, PbI_2) from Alfa Aesar (Tewksbury, MA USA). Borosilicate glass coverslips (Fisherbrand® 1250C, 25×25×0.22 mm) were purchased from Fisher Scientific (Pittsburgh, PA, USA).

Methods

Synthesis

Perovskite thin films were synthesized via solvent-washing, spin-coating method and protected by a polystyrene layer as described previously.[1] As synthesized films $MAPb(Br_xI_{1-x})_3$ will further be noted by their nominal composition *x*. Samples for X-ray Diffraction (XRD) did not have a polystyrene layer. Samples used in scanning electron microscopy (SEM) were fabricated without a polystyrene layer on top of a silicon wafer substrate instead of glass and one sputtered with gold for the determination of pinhole size. For atomic force microscopy (AFM) measurements a sample on a silicone substrate was used.

Ellipsometry Measurements

Ellipsometry measurements were carried out on a VASE® ellipsometer from J.A. Woollam Co. (Lincoln, NE USA). For modelling and fitting the included software WVASE® was used. All fits were evaluated by the mean square error (MSE) with a Marquardt-Levenberg algorithm in which the change in MSE was lower than 10^{-10}. Oscillator models using eight Tauc-Lorentz oscillators for the dielectric function of the perovskites were either taken directly from literature[2] or created by linearly fitting the oscillator parameters of dielectric functions of x=0.25, x=0.5, and x=0.75 from literature and adjusting the parameters further to fit critical points.[3–5] Critical points were determined from the second derivative of the dielectric function for all functions listed in Fujimoto et.al[2] and interpolated to the composition used. The imaginary and real component of the dielectric functions are described by the following formulas with E_g being the optical gap, E_o the peak transition energy, A the amplitude, and C the broadening of a Tauc-Lorentz Peak, and $\varepsilon_1(\infty)$ a constant contribution at high energies[6].

$$\varepsilon_1 = \sum_{j=1}^{m}(\varepsilon_{1,j}(\infty) + \frac{2}{\pi}P\int_{E_g}^{\infty}\frac{E'\varepsilon_{2,j}(E')}{E'^2-E^2}dE') \qquad (1)$$

$$\varepsilon_2 = \sum_{j=1}^{m}\frac{A_jC_jE_{0,j}(E-E_{g,j})^2}{E\left[(E^2-E_{0,j}^2)^2+C_j{}^2E^2\right]} \text{ for } E > E_{g,j} \text{ and } \varepsilon_2 = 0 \text{ for } E \leq E_{g,j} \qquad (2)$$

Created models were validated against several samples with the corresponding composition and further adjusted if needed at angles 60, 65, 70, 75 °, as well as transmission measurements, between 300-1200 nm in 5 nm steps. For a list of all oscillator parameters used see **Table S1**. The imaginary part of all dielectric functions and critical points used are shown in **Figure S1**. The cleaned glass coverslips were measured in the same energy range by themselves and modeled with a Cauchy layer as described by equation (3), λ being the wavelength. For the polystyrene layer a model provided by the ellipsometer manufacturer and validated against polystyrene samples on the same coverslips was used. It consists of two Gaussian and one Tauc-Lorentz peak resulting in the imaginary part of the dielectric function as described by equation (4).

$$n = 1.5035 + \frac{0.0076854\mu m^2}{\lambda^2} + \frac{-9.3589\cdot10^{-5}\mu m^4}{\lambda^4} \text{ and } k = 5.2627\cdot10^{-6}\cdot e^{8.5604(1.24\mu m(\frac{1}{\lambda}-\frac{1}{340\,nm}))} \qquad (3)$$

$$\varepsilon_2 = 0.93253eV\left(e^{-\left(\frac{E-5.6743eV}{0.1643eV}\right)^2} - e^{-\left(\frac{E+5.6743eV}{0.1643eV}\right)^2}\right) + 0.06696eV\left(e^{-\left(\frac{E-4.9198eV}{0.4657eV}\right)^2} - e^{-\left(\frac{E+4.9198eV}{0.4657eV}\right)^2}\right) + \frac{167.69eV\cdot0.81946eV\cdot6.1021eV(E-5.3187eV)^2}{E[(E^2-(6.1021eV)^2)^2+(0.81946eV)^2E^2]} \qquad (4)$$

For segregation measurements samples of composition x=0.5 were measured at 60, 65, 70, 75 ° between 400-1200 nm in 5 nm steps and at 50 ° between 500-800 nm in 1 nm steps. Thickness, as well as the number of backside reflections resulting from the use of a transparent substrate and thickness non-uniformity were fitted before illumination and if necessary further adjusted to fit the observed peak. For this a model with layers glass, perovskite, and polystyrene was used. No interface layers in between the perovskite and other layers were used because additional fittings showed that they have negligible effects on our fittings of these samples. The results of fits for three samples each measured three times are shown in **Table S2**. Dynamic measurements under illumination and during recovery in the dark were tracked via a peak of Ψ at a wavelength between 600 and 650 nm including the values at 5 nm and 10 nm lower and higher. The exact wavelengths examined were different for each sample. Time between measurements of the same wavelength was approximately 38 s. The illumination was carried out with a green laser (532 nm wavelength) at 11 mW/cm² in a circular area of about 2.5 mm in diameter. Recovery in the dark was guaranteed by enclosing the setup in a dark box.

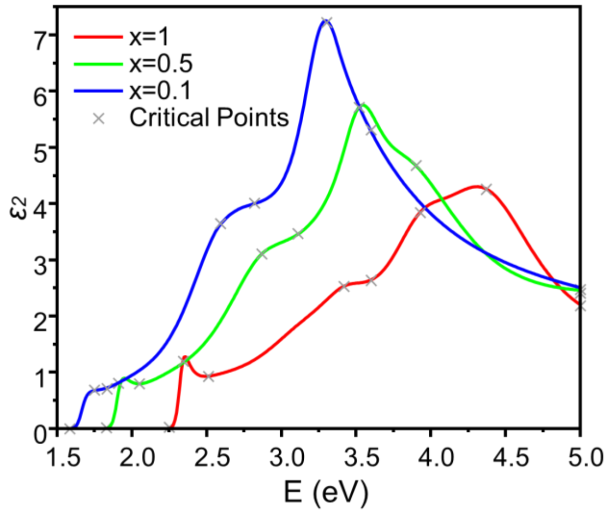

Figure S1. Imaginary part of the dielectric functions used for ellipsometric fits.

Table S1. Oscillator parameters for dielectric functions of mixed halide perovskites.

	Oscillator	1	2	3	4	5	6	7	8
x=1	*Eg* in eV	2.25	2.25	2.25	2.25	2.25	2.25	2.25	2.25
	Eo in eV	2.33	2.34	3.1	3.42	3.92	4.36	5.88	9.65
	A in eV	70.13	30.02	8.69	2.76	5.03	11.13	4.7	18.53
	C in eV	0.09	0.6	0.83	0.47	0.58	0.9	1.41	0.77
	$\varepsilon_1(\infty)$	1							
x=0.5	*Eg* in eV	1.827	1.847	1.881	2.032	2.895	2.869	3.405	4.365
	Eo in eV	1.843	1.903	1.993	2.864	3.198	3.509	3.791	4.366
	A in eV	14.467	52.571	24.25	24.333	60.788	26.191	57.244	142.483
	C in eV	0.083	0.1	0.838	0.889	0.822	0.377	0.62	4.84
	$\varepsilon_1(\infty)$	1.283							
x=0.1	*Eg* in eV	1.587	1.617	1.677	1.818	2.594	2.661	3.377	3.910
	Eo in eV	1.607	1.659	1.719	2.584	2.639	3.256	3.337	3.882
	A in eV	6.139	40.404	23.230	24.207	103.922	45.541	161.650	68.038
	C in eV	0.076	0.120	0.743	0.745	1.0048	0.412	0.337	6.612
	$\varepsilon_1(\infty)$	1.2413							

Table S2. Fitting results of samples with *x*=0.5.

Sample	1	2	3
Thickness Perovskite in nm	197.1 ± 0.7	194 ± 6	193 ± 3
Thickness Polystyrene in nm	1036 ± 5	1034 ± 13	1036 ± 3
Thickness non-uniformity in %	6.4 ± 0.2	5 ± 1	7 ± 1
Number of Backside reflections	0.39 ± 0.01	0.65 ± 0.01	0.72 ± 0.02

Additional Measurements

Photoluminescence (PL) measurements were carried out at the same wavelength and power density as ellipsometry measurements using a homebuilt PL emission microspectroscope / spectro-microscope described in a previous work.[1] Unless otherwise specified one plot is created from the sum of 60 frames with 0.1 s exposure time each. UV-Vis measurements were done with a halogen light source and UV-Vis LR-1 (ASEQ instruments, Vancouver, Canada).

Atomic Force Microscopy (AFM) measurements were carried out in contact mode with a silicon nitride AFM tip (PNB-DB-20, Nanoworld technologies) on a MFP 3D microscope (Asylum Research). Thin scratches were made on the film with a

sharp needle before measuring the height profiles of scratched and intact parts of the film. In each of three different locations on the sample two thickness measurements of the same scratch with a distance of about 30 µm were considered. Thickness non-uniformity was estimated from the differences between two lines in the same picture.

For XRD measurements a Rigaku MiniFlex-II with a Cu anode, 30 kV, and 15 mA tube was used. One sample was measured between 10 ° and 35 ° in 0.1 ° steps. SEM measurements were carried out on a Hitachi 4500 at 20 kV and 10 µA. For the domain size analysis the software ImageJ[7] was used. The contrast was enhanced, the domains were drawn over with a paintbrush tool, a threshold was set so that the borders of domains were white and the inside dark, and the "analyze particle" tool used to determine the area of the domains. Their size was then estimated assuming circular domains.

Supplementary Figures and Tables

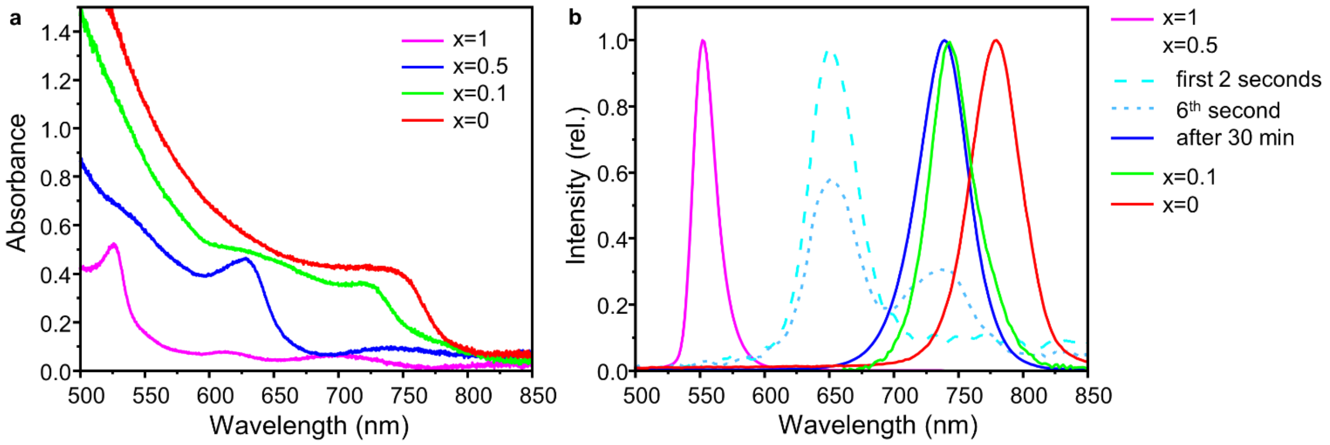

Figure S2. a) UV-Vis spectroscopy and b) photoluminescence of different perovskite compositions $x = 1$ (purple), 0.5 (blue), 0.1 (green), and 0 (red). Time snapshots for $x = 0.5$ emission are shown in (b). Plots for x=0.5 for first 2 seconds and 6th second are created from the sum of frame 0-20 and 50-60 of one measurement respectively with the integration time of each frame 100 ms using our spectro-microscopy calibrated with noble gas lamps.[1] We can see that the phase segregated evidenced by peak changing and shifting over time. The rest emission curves are averaged from the first 6 s of excitation light exposure and peaks are normalize to one.

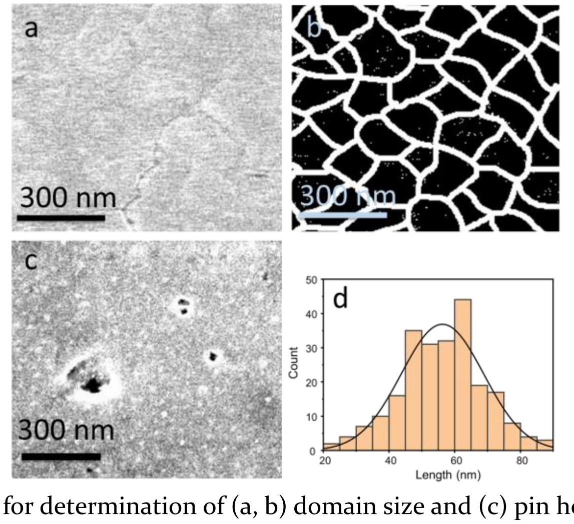

Figure S3. SEM of sample with x=0.5 for determination of (a, b) domain size and (c) pin hole size with (d) histogram of domain size. The perovskite film looks glassy flat with eye and uniform under an optical microscope. Domain boundaries and pin holes can be seen under the SEM.

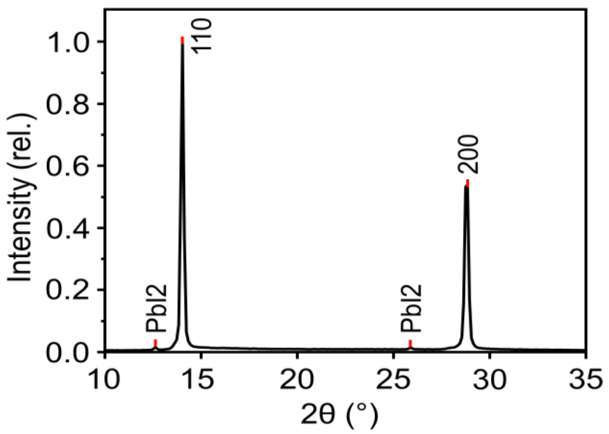

Figure S4. XRD of sample with composition *x*=0.5.

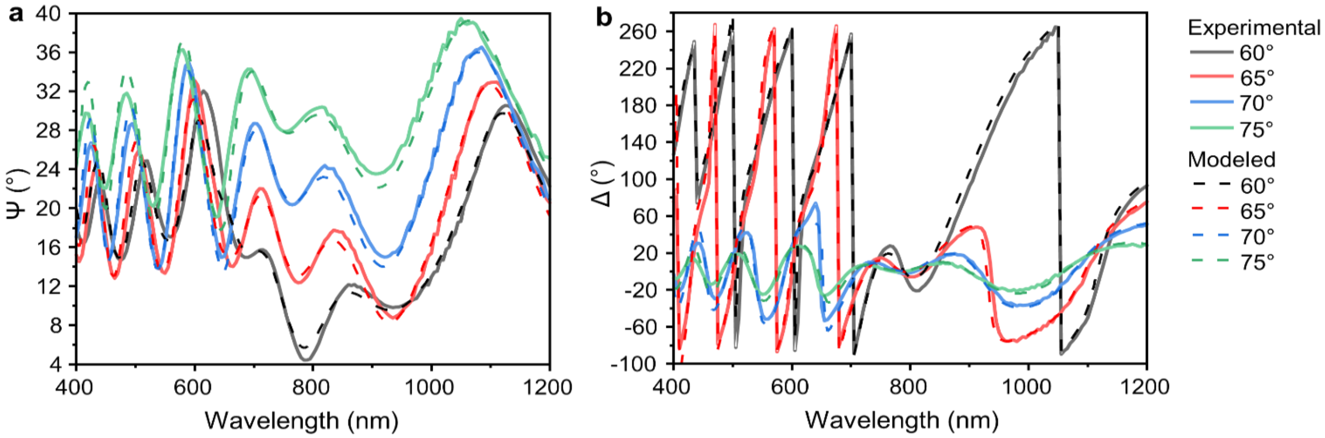

Figure S5. Ellipsometric measurement and modelling of *x*=0.5 of a) Ψ and b) Δ.

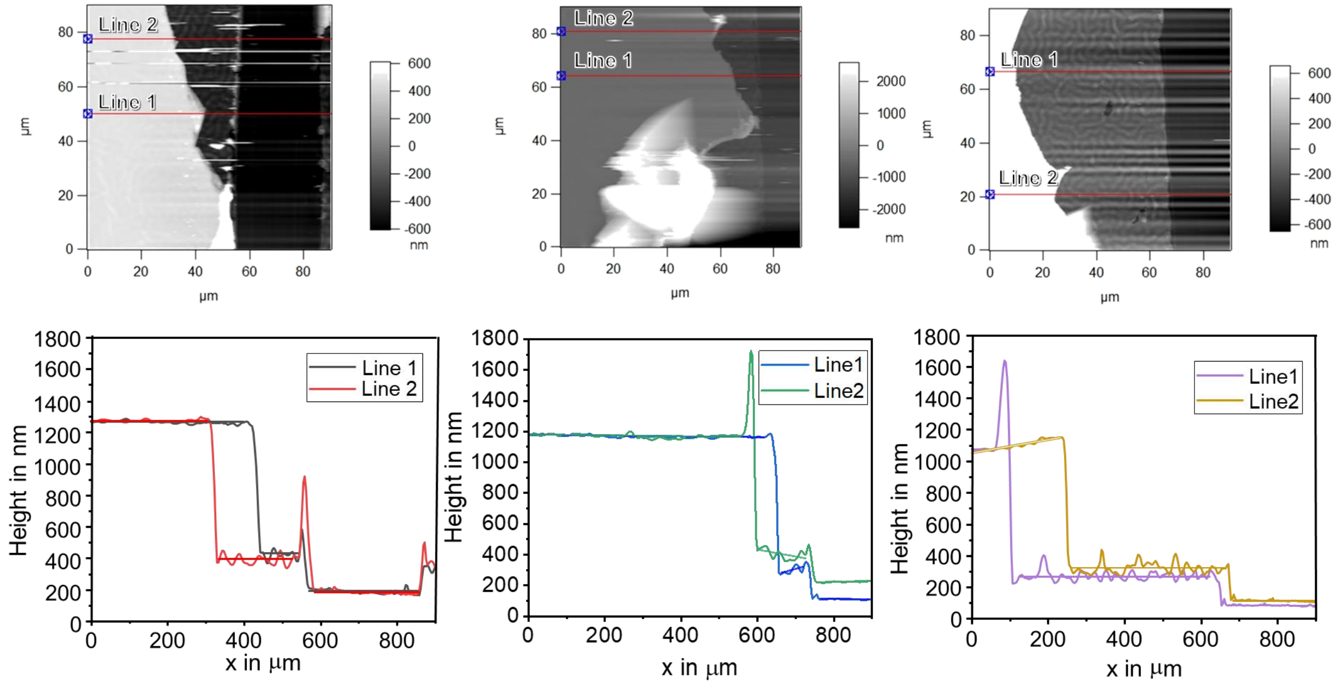

Figure S6. (Upper panel) example AFM images with polymer and perovskite films scratched broken after ellipsometric measurements and (lower panel) example cross-sections. The scratches on the right sides of the images (dark areas) are created vertically by a needle and the lowest (darkest) layers are the silicon substrate, the middle layers are the perovskite thin film, and the top layers are the polymer coating film. Color bar showing z-axis with center auto scaled by the AFM software, i.e. only the relative colors matter. Horizontal white lines on the AFM images are caused by the debris of the scratching that are big and mobile. They do not affect the estimation of the thickness at the locations that are clean (red lines). The uniform polymer and perovskite films are scratched after the ellipsometry measurements with a few vertical scratches with width in the order of 1 mm for AFM measurements. The silicon surface is too hard to scratch and no damage on its surface is observed. There is plenty of film available for further ellipsometry measurements. The cross-sections are shown in the lower panel with each plateau represents the surface of the polymer, perovskite, and silicon from left to right across the scratches on the substrate. The bumps on the perovskite surface are likely to be polymer debris because bare perovskite surface is smooth under AFM and SEM. The thicknesses are averaged from the AFM measurements on three different locations of the film by taking the height difference between the surfaces as the thickness, i.e. ~800 nm for the polymer and ~200 nm for the perovskite film thickness from these cross-section curves both are consistent with the ellipsometric measurements.

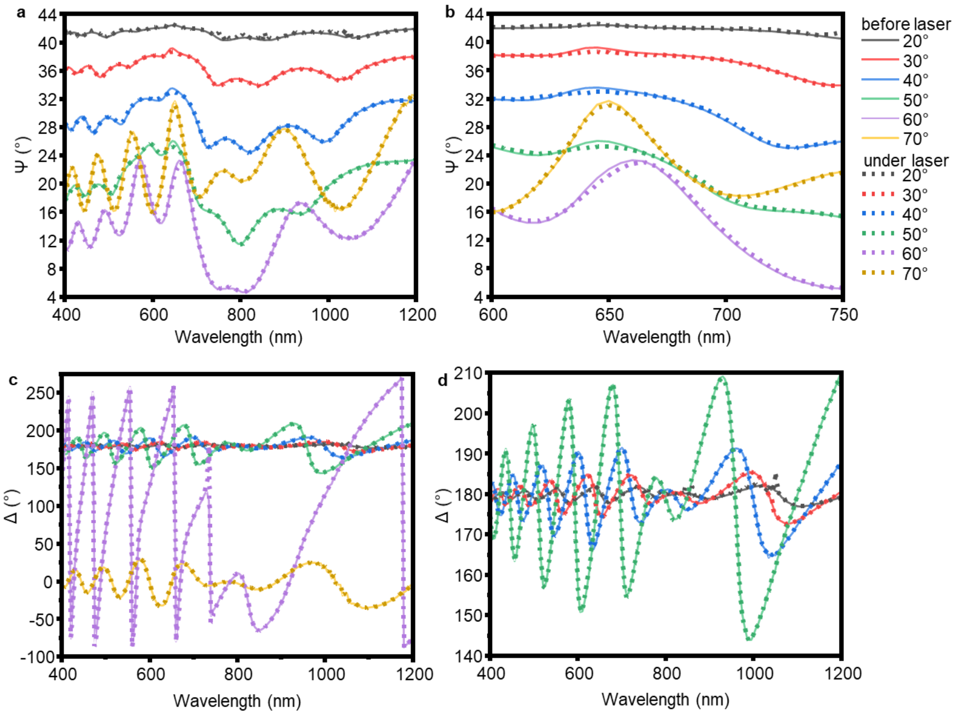

Figure S7. Ellipsometric measurements of x=0.5 at different angles 20 – 70 ° of a) Ψ over whole range, b) relevant peak in Ψ, c) Δ at all angles, and d) Δ at angles 20 – 50 °.

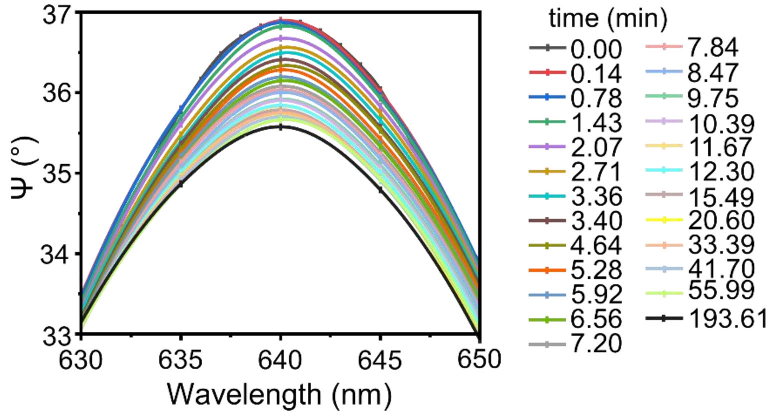

Figure S8. Time labeling of Figure 2b in the main text.

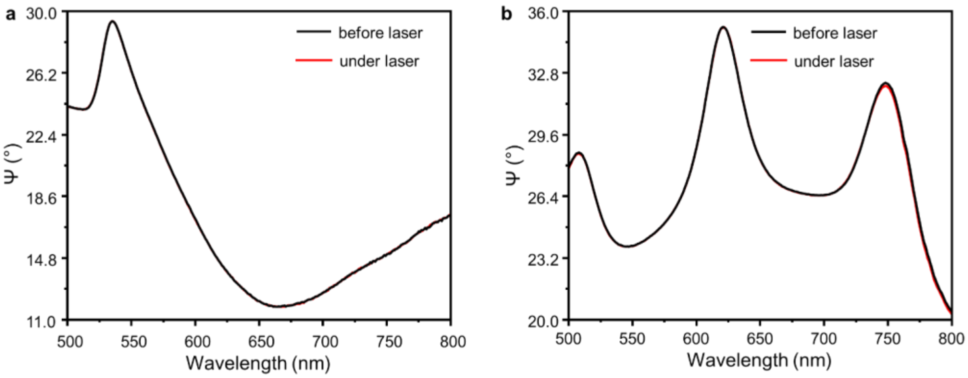

Figure S9. Ellipsometric measurements of Ψ at 50 ° of a) x=1 and b) x=0.1.

Table S3. Time constants k of all measurements in 10^{-3} s^{-1} from single exponential model: $p_{0.5} = A \cdot e^{-kx} + p_{0.5_0}$.

Sample	1			2			3		
Segregation	1.2	1.8	2.8	2.2	0.2*	2.0	1.1	1.8	0.8
Recovery	0.12	0.17	0.09	0. 28	0. 10	0. 16	0. 11	0. 14	0. 18

* outlier left out in average and standard deviation calculation

Table S4. Measured endpoint composition of all measurements represented by $p_{0.5}$ in %.

Sample	1			2			3		
Segregation	74	75	70	84	81	74	76	76	82
Recovery	101	97	96	97	104	99	100	99	99

Table S5. Measurement times of all measurements in min.

Sample	1			2			3		
Segregation	118	117	194	107	112	138	183	117	108
Recovery	1205	1610	1210	1200	1055	1260	1245	1335	1260

Table S6. Parameters of all recovery measurements from double exponential model: $p_{0.5} = A_1 \cdot e^{-k_1 x} + A_2 \cdot e^{-k_2 x} + p_{0.5_0}$.

Sample	1			2			3			Average ± Standard deviation
A_1 in %	$-3 \cdot 10^5$	$-2 \cdot 10^{10}$	$-2 \cdot 10^{10}$	$-3 \cdot 10^5$	$-1 \cdot 10^{15}$	$-1 \cdot 10^4$	$-1 \cdot 10^6$	$-8 \cdot 10^2$	$-2 \cdot 10^5$	$-2 \cdot 10^{14} \pm 5 \cdot 10^{14}$
k_1 in 10^{-3} s^{-1}	1.41	3.33	1.97	1.47	4.80	0.969	0.989	2.64	1.54	2.12 ± 1.27
A_2 in %	-19	-4	-35	-17	-15	-17	-18	-18	-17	-18 ± 8
k_2 in 10^{-3} s^{-1}	0.08	0.13	0.09	0.11	0.08	0.09	0.05	0.09	0.12	0.09 ± 0.02
$p_{0.5_0}$ in %	100	97	100	97	104	99	96	99	99	99 ± 2
$k_{average}$* in 10^{-3} s^{-1}	1.40	1.46	0.99	3.33	4.80	1.66	1.97	0.95	1.54	2.01 ± 1.26

*Amplitude weighted average calculated as $k_{average} = {t_{average}}^{-1}$ with $t_{average} = \left(\frac{A_1 t_1 + A_2 t_2}{A_1 + A_2}\right)$

References

(1) Vicente, J. R.; Kordesch, M. E.; Chen, J. Stabilization of mixed-halide lead perovskites under light by photothermal effects. *Journal of Energy Chemistry* **2021**, *63*, 8–11. DOI: 10.1016/j.jechem.2021.08.046.

(2) Fujimoto, S.; Fujiseki, T.; Tamakoshi, M.; Nakane, A.; Miyadera, T.; Sugita, T.; Murakami, T. N.; Chikamatsu, M.; Fujiwara, H. Organic-Inorganic Hybrid Perovskites. In *Spectroscopic Ellipsometry for Photovoltaics*; Fujiwara, H., Collins, R. W., Eds.; Springer Series in Optical Sciences; Springer International Publishing, 2018; pp 471–493. DOI: 10.1007/978-3-319-95138-6_10.

(3) Minoura, S.; Maekawa, T.; Kodera, K.; Nakane, A.; Niki, S.; Fujiwara, H. Optical constants of Cu(In,Ga)Se2 for arbitrary Cu and Ga compositions. *Journal of Applied Physics* **2015**, *117* (19), 195703. DOI: 10.1063/1.4921300.

(4) Snyder, P. G.; Woollam, J. A.; Alterovitz, S. A.; Johs, B. Modeling AlxGa1−xAs optical constants as functions of composition. *Journal of Applied Physics* **1990**, *68* (11), 5925–5926. DOI: 10.1063/1.346921.
(5) Yao, H.; Woollam, J. A.; Wang, P. J.; Tejwani, M. J.; Alterovitz, S. A. Spectroscopic ellipsometric characterization of Si/Si1−xGex strained-layer supperlattices. *Applied Surface Science* **1993**, *63* (1-4), 52–56. DOI: 10.1016/0169-4332(93)90063-H.
(6) Nakane, A.; Fujimoto, S.; Jellison, G. E.; Herzinger, C. M.; Hilfiker, J. N.; Li, J.; Collins, R. W.; Koida, T.; Kim, S.; Tampo, H.; Fujiwara, H. Inorganic Semiconductors and Passivation Layers. In *Spectroscopic Ellipsometry for Photovoltaics*; Fujiwara, H., Collins, R. W., Eds.; Springer Series in Optical Sciences; Springer International Publishing, 2018; pp 319–426. DOI: 10.1007/978-3-319-95138-6_8.
(7) Schneider, C. A.; Rasband, W. S.; Eliceiri, K. W. NIH Image to ImageJ: 25 years of image analysis. *Nature methods* **2012**, *9* (7), 671–675. DOI: 10.1038/nmeth.2089.
